# Modification of Cul1 regulates its association with proteasomal subunits

**DOI:** 10.1186/1747-1028-1-5

**Published:** 2006-04-28

**Authors:** Joanna Bloom, Angelo Peschiaroli, George DeMartino, Michele Pagano

**Affiliations:** 1 Department of Pathology, New York University Cancer Institute and New York University School of Medicine, New York 10016, USA; 2 Department of Physiology, University of Texas Southwestern Medical Center, Dallas, Texas 75235, USA; 3 The Rockefeller University, New York 10021, USA

## Abstract

**Background:**

Ubiquitylation targets proteins for degradation by the 26S proteasome. Some yeast and plant ubiquitin ligases, including the highly conserved SCF (Skp1/Cul1/F-box protein) complex, have been shown to associate with proteasomes. We sought to characterize interactions between SCF complexes and proteasomes in mammalian cells.

**Results:**

We found that the binding of SCF complexes to proteasomes is conserved in higher eukaryotes. The Cul1 subunit associated with both sub-complexes of the proteasome, and high molecular weight forms of Cul1 bound to the 19S proteasome. Cul1 is ubiquitylated *in vivo*. Ubiquitylation of Cul1 promotes its binding to the S5a subunit of the 19S sub-complex without affecting Cul1 stability.

**Conclusion:**

The association of ubiquitylating enzymes with proteasomes may be an additional means to target ubiquitylated substrates for degradation.

## Background

The significance of ubiquitylation for the proteasomal degradation of many proteins has been well established. Studies using cell-lines that are temperature-sensitive for the E1 ubiquitin-activating enzyme indicate that the bulk of proteasomally degraded proteins accumulate at the non-permissive temperature, suggesting that ubiquitylation is necessary for their proteolysis [1]. However, whether ubiquitylation is the sole signal for recognition and subsequent degradation of substrates by the 26S proteasome is still unclear. The S5a subunit of the 19S proteasome (called Rpn10 in yeast) has affinity for ubiquitin chains [2]; however, the19S subunit Rpt5/S6' has been shown to recognize ubiquitylated proteins [3]. Deletion of *rpn10 *in yeast does not disrupt the degradation of the majority of short-lived proteins [4], suggesting there is some redundancy in targeting ubiquitylated substrates for degradation. It is possible that additional proteasomal subunits are capable of recognizing ubiquitylated substrates, and/or other cellular factors may be involved in the delivery of substrates from the ubiquitylation machinery to the proteasome (reviewed in [5]).

Several studies have shown that the ubiquitylation of specific substrates is coupled to degradation by the 26S proteasome through the interaction of their respective ubiquitin ligases (E3s) with the proteasome. In particular, two E3s in yeast, Ubr1 and Ufd4, have been shown to associate with subunits of the regulatory complex of the proteasome, and these interactions appear to be direct [6]. Importantly, if the proteasome-binding site in Ufd4 is deleted while the region responsible for ubiquitin ligation is left intact (Ufd4-ΔN), substrates of Ufd4 are efficiently ubiquitylated but are no longer degraded [6]. This indicates that the interaction of Ufd4 with the proteasome is involved in the degradation of its ubiquitylated substrates. The existence of two signals that contribute to recognition by the proteasome, that is, the ubiquitylation of the substrate and the association of the ubiquitin ligase with the proteasome, would be expected to increase both the specificity and efficiency that is fundamental to the ubiquitin-proteasome pathway. Ufd4 and perhaps other ubiquitin ligases may have a dual role in the ubiquitin-proteasome pathway; they contribute both to substrate ubiquitylation and to proteasome-targeting. This may account for the only partial defect in proteolysis observed in *rpn10*-deleted yeast cells. In fact, overexpression of wild-type Ufd4 in *rpn10*-deleted cells rescued the degradation of ubiquitylated forms of an engineered β-galactosidase, Ub76-V-βgal, which contains a "non-removable" ubiquitin moiety, while Ufd4-ΔN did not have this effect [6]. Two signals that contribute to proteasome-targeting may also explain why some proteins that have been engineered to preclude ubiquitylation are still proteasomally degraded. In the absence of polyubiquitin chain formation, these substrates may be delivered directly to the proteasome through their association with their respective ubiquitin ligases.

SCF complexes represent a large family of ubiquitin ligases, and are so called, because they are composed of Skp1, Cul1, an F-box protein and Roc1. It is the F-box protein (FBP) component of the SCF complex that is directly responsible for substrate recognition (reviewed in [7]). In yeast, Cdc53 (the ortholog of Cu11), Skp1, and the SCF-associated E2, Cdc34 were found to be associated with affinity-purified proteasomes [8]. An interaction between Skp1 and the proteasome has also been described in *Arabidopsis *[9]. A yeast two-hybrid screen identified both the *Arabidopsis *orthologue of Skp1 and the alpha 4 proteasome subunit as interactors of *Arabidopsis *SnRK proteins. The SnRK proteins enhance the binding of Skp1 to alpha 4, and SnRK, Skp1 and Cul1 can be co-purified with proteasomes. The yeast-related SnRK protein, Snf1, is a kinase involved in glucose-mediated transcriptional regulation [10]. Interestingly, the association of *Arabidopsis *SnRK with PRL, a protein that inhibits SnRK kinase activity *in vitro*, diminishes the binding of SnRK to Skp1 and may, therefore, prevent the association of SCF complexes with proteasomes in *Arabidopsis *[9]. This study suggests that the function of SnRKs in the context of proteasome-binding and Skp1-binding may be altogether different than their role in transcriptional regulation in yeast.

We sought to determine if mammalian SCF complexes, similarly to other ubiquitin ligases, bind to subunits of the 26S proteasome. Following is a description of our results.

## Results and discussion

### Co-immunoprecipitation of Cul1 and other SCF components with proteasome subunits

**Figure 1:**
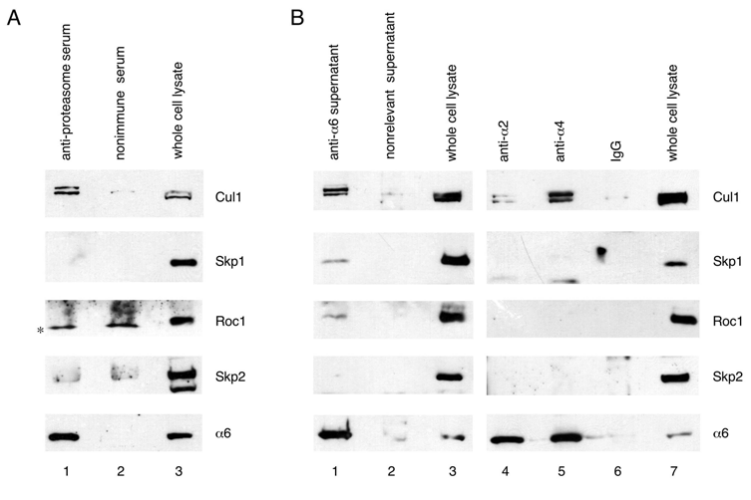
**Co-immunoprecipitation of SCF subunits with proteasome components**. (A) Extracts from Hela cells were immunoprecipitated with a polyclonal antibody that recognizes multiple proteasomal subunits (lane 1) or a nonrelevant polyclonal serum (lanes 2). Lane 3 contains whole cell lysate. Immunoprecipitates were analyzed by immunoblotting with antibodies to the indicated proteins. A non-specific cross-reactive band is indicated by the asterisk. (B) Extracts from Hela cells were immunoprecipitated with a monoclonal antibody to alpha 6 (lane 1), a monoclonal antibody to alpha 2 (lane 4), a monoclonal antibody to alpha 4 (lane 5), a nonrelevant supernatant (lane 2) or mouse IgG (lane 6). Lanes 3 and 7 contain whole cell lysate. Immunoprecipitates were analyzed by immunoblotting with antibodies to the indicated proteins.

Since physical interactions between SCF components and proteasomes have been observed in yeast [8] and *Arabidopsis *[9], we sought to determine if SCF subunits were associated with proteasomes in mammalian cells. We first tested if antibodies to proteasome subunits could co-precipitate SCF subunits from cells. Lysates from HeLa cells were subjected to immunoprecipitation with a polyclonal antibody that recognizes multiple subunits of both the 19S and 20S proteasome, and associated proteins were analyzed by immunoblotting. We found that Cul1, but not Skp1, Roc1 or Skp2 coprecipitated with the proteasome under these conditions (Figure 1A). We then used monoclonal antibodies to the alpha 6, alpha 2 and alpha 4 subunits of the 20S proteasome to specifically immunoprecipitate these subunits from HeLa cell lysate. Cul1 co-immunoprecipitated with the alpha 6, alpha 2 and alpha 4 subunits (Figure 1B). In addition, some Skp1 and Roc1 were also present in the alpha 6 immunoprecipitate (Figure 1B, lane 1). It is possible that the Cul1 observed in these immunoprecipitates is bound to proteasomal subunits other than the alpha subunits but still coprecipitates with alpha subunits. These results suggest that Cul1 binds to the 26S proteasome and can bind to alpha subunits of the 20S proteasome, although it is not clear at this time whether this interaction is direct. These results demonstrate that similarly to yeast and plant SCF complexes, mammalian SCF complexes are associated with proteasomes.

### The N-terminus of Cul1 binds alpha subunits of the 20S proteasome

**Figure 2:**
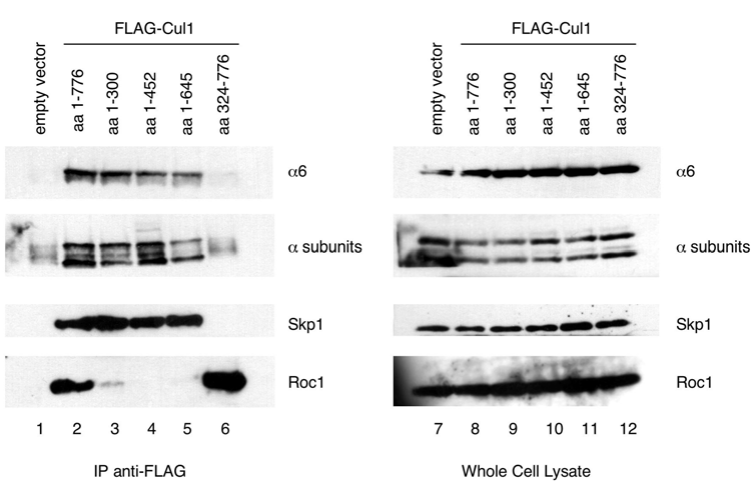
**The N-terminus of Cul1 binds alpha subunits of the 20S proteasome**. 293T cells were transfected with empty vector (lanes 1 and 7) or FLAG-tagged versions of full-length Cul1 aa 1-776 (lanes 2 and 8), Cul1 aa 1-300 (lanes 3 and 9), Cul1 aa 1-452 (lanes 4 and 10), Cul1 aa 1-645 (lanes 5 and 11) or Cul1 aa 324-776 (lanes 6 and 12). Extracts were immunoprecipitated with a monoclonal antibody to the FLAG epitope (lanes 1–6). Lanes 7–12 contain whole cell lysates from transfected cells. Immunoprecipitates and whole cell lysates were analyzed by immunoblotting with antibodies to the indicated proteins.

To determine which region of Cul1 is responsible for binding to the proteasome, we overexpressed and immunoprecipitated deletion mutants of Cul1 and associated proteins. 293T cells were transfected with FLAG-tagged versions of full-length Cul1 (aa 1-776) and several Cul1 mutants including, Cul1 (aa 1-300), Cul1 (aa 1-452), Cul1 (aa 1-645) and Cul1 (aa 324-776). Extracts from transfected cells were immunoprecipitated with an antibody to the FLAG epitope, and associated proteins were analyzed by immunodetection with an antibody to the alpha 6 subunit specifically as well as an antibody that recognizes multiple alpha subunits. We found that full-length Cul1 and the deletion mutants of Cul1 that contained the N-terminal region co-immunoprecipitated alpha subunits of the 20S proteasome, while the mutant encoding the C-terminus of Cul1 (aa 324-776) did not (Figure 2). These results demonstrate that amino acids 1–300 are necessary and sufficient for the interaction of Cul1 with 20S proteasomes.

We also looked for the presence of proteins whose binding to Cul1 has already been mapped. As expected, Skp1 bound full-length Cul1 and the N-terminal mutants, while Roc1 bound full-length Cul1 and the C-terminal mutant. In additional experiments, we found that amino acids 1–150 or 150–400 of Cul1 alone are not sufficient to co-precipitate proteasome subunits (data not shown). We are therefore unable to ascertain at this time if the portion of Cul1 interacting with the proteasome overlaps with the binding site for Skp1.

Our finding that the N-terminus of Cul1 is sufficient for proteasome-binding indicates that SCF complexes are not merely anchored to proteasomes via the interaction of the associated ubiquitylated substrate with the 19S subcomplex. This Cul1 deletion mutant associates with Skp1 and F-box proteins, but not the ubiquitylation machinery, and, as a result, SCF substrates are bound, but not ubiquitylated. Similarly, yeast Cul1 can bind to the proteasome in the absence of a functional ubiquitin-conjugating enzyme (*cdc34ts*) [8]. Taken together, these results suggest alternative models for the association of SCF complexes with proteasomes. One possibility is that SCF complexes are permanently bound to proteasomes, and substrates are captured by the appropriate F-box protein and then rapidly ubiquitylated and degraded. This model seems improbable since substrates must contact their respective F-box proteins in a spatially and temporally regulated manner and this model would rely on diffusion of substrates and proteasomes. An alternative model is that Cul1 is permanently associated with proteasomes, and Skp1/F-box proteins transiently associate after recognition of the appropriate substrate for coupled ubiquitylation and degradation. Structural studies of Skp1 and Skp2 indicate that these two proteins form a very stable complex [11], and it is possible that this analysis can be extended to other F-box proteins. Finally, there exists a model in which entire SCF complexes exist in proteasome-bound and unbound states. The findings in *Arabidopsis *indicate that Skp1 can bind to the proteasome with high affinity only in the presence of SnRK. SnRk binding to Skp1 is, in turn regulated by its association with PRL [9]. A similar system may exist in mammalian cells, in which SCF binding to the proteasome is regulated by its ability to bind an additional factor mediating the association of SCF complexes with the proteasome. This would provide an additional level of regulation for proteasome-binding that has not yet been characterized.

### Co-purification of SCF subunits with 26S, 19S and 20S proteasomes

**Figure 3:**
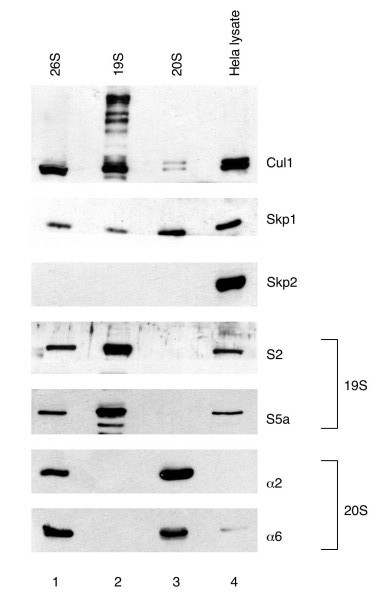
**Co-purification of SCF subunits with proteasomes**. Lane 1 contains purified 26S proteasomes, lane 2 contains purified 19S proteasomes, lane 3 contains purified 20S proteasomes and lane 4 contains whole cell lysate from Hela cells. Purified proteasomes and protein extracts were analyzed by immunoblotting with the antibodies to the indicated proteins.

To confirm that SCF complexes were associated with proteasomes, we determined if components of SCF ligases were present in purified 26S, 19S and 20S proteasome preparations. Western blot analysis was performed on 26S, 20S and 19S proteasomes purified from bovine red blood cells provided by Dr. G. DeMartino [12-14]. We found that Cul1 and Skp1 were abundant in all three proteasome preparations, while the F-box protein Skp2 was completely absent (Figure 3). Since bovine and human Skp2 are highly conserved (399 of 424 amino acids are identical), it is unlikely that the absence of a Skp2 signal in the purified proteasomes is due to an inability of the antibody to recognize bovine Skp2. Other F-box proteins tested (Fbl3 and βTrCP1) were also absent from 26S proteasomes (data not shown). Since Cul1 and Skp1 can associate with numerous F-box proteins, it is possible that the association of F-box proteins with proteasomes is more difficult to detect. Therefore, we cannot rule out the possibility that complete SCF complexes are bound to proteasomes. To ensure the purity of 19S and 20S preparations, immunodetection was carried out using antibodies specific to 20S subunits alpha 2 and alpha 6 and antibodies specific to the 19S subunits S2 and S5a. Similar results were obtained using purified 26S proteasomes from Dr. M. Rechsteiner (data not shown).

Interestingly, we observed high molecular weight forms of Cul1 in 19S proteasomes (Figure 3, lane 1). We reasoned that these forms could represent ubiquitylated or neddylated Cul1. Polyneddylated forms of Cul1 have been observed *in vitro *[15]. Our results suggest the possibility that post-translational modification of Cul1 enhances its association with subunits of the 19S proteasome.

### Ubiquitylated forms of Cul1 bind the S5a subunit of the 19S proteasome *in vitro*

**Figure 4:**
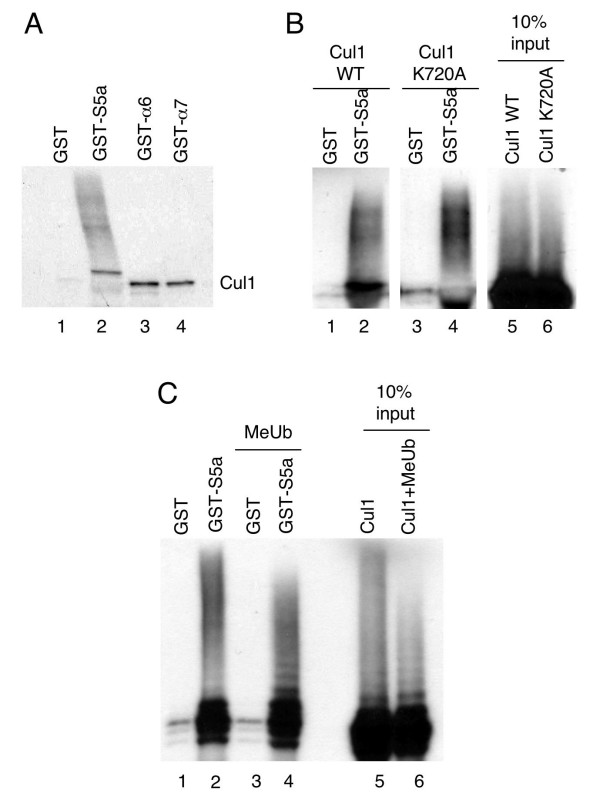
**Ubiquitylated Cul1 binds the S5a subunit of the 19S proteasome**. (A) Pulldown of ^35^S Cul1 using GST alone (lane 1), GST-S5a (lane 2), GST-αlpha 6 (lane 3) or GST-αlpha 7 (lane 4). (B) Pulldown of ^35^S wild-type Cul1 (lanes 1 and 2) or ^35^S Cul1(K720A) (lanes 3 and 4) using GST alone (lanes 1 and 3) or GST-S5a (lanes 2 and 4). Ten percent input of ^35^S wild-type Cul1 and ^35^S Cul1(K720A) are shown in lanes 5 and 6, respectively. (C) Pulldown of ^35^S wild-type Cul1 *in vitro *translated in the absence (lanes 1 and 2) or presence (lanes 3 and 4) of methylated ubiquitin (MeUb) using GST alone (lanes 1 and 3) or GST-S5a (lanes 2 and 4). Ten percent input of ^35^S Cul1 *in vitro *translated in the absence or presence of MeUb are shown in lanes 5 and 6, respectively.

Since previous work has demonstrated that the S5a subunit of the 19S proteasome has high affinity for ubiquitin chains [2], we tested whether high molecular weight forms of Cul1 could bind S5a *in vitro*. Cul1 was *in vitro *translated using rabbit reticulocyte lysate in the presence of ^35^S methionine and tested for its ability to bind to beads coated with GST-tagged proteasomal subunits S5a, alpha 6 and alpha 7. We found that high molecular weight forms of Cul1 bound to S5a (Figure 4A, lane 2). This result is consistent with our observation that high molecular weight forms of Cul1 co-purify with 19S proteasomes (Figure 3). The unmodified form of Cul1 associated with GST-tagged alpha 6 and alpha 7 (Figure 4A, lanes 3 and 4). This is in accordance with our results showing that Cul1 binds alpha subunits of the 20S proteasome (Figures 1 and 2). Since rabbit reticulocyte lysate contains both proteasomes and ubiquitylating enzymes [16], this result does not yet clarify whether the binding of Cul1 to the 20S proteasome is direct or indirect.

It has been reported that Cul1 can be polyneddylated [15] and that Nedd8 conjugates can be targeted to the S5a subunit of the proteasome through the adaptor molecule Nub1 [17]. We therefore tested whether the high molecular weight forms of Cul1 that bind to GST-S5a are polyneddylated forms of Cul1. To do this, we assayed the ability of *in vitro *translated Cul1 that is mutated in its neddylation site (Cul1K720A) to bind GST-S5a (Figure 4B). Although it is not clear if Cul1 is polyneddylated at K720 or if Cul1 is mononeddylated at multiple sites, the K720 residue is required for neddylation to occur [15]. We found the high molecular weight forms of Cul1 bound to GST-S5a whether or not the neddylation site of Cul1 was intact (Figure 4B, compare lanes 2 and 4), indicating that the forms of Cul1 binding to S5a are not polyneddylated forms of Cul1. Accordingly, Cdc53 associated with proteasomes in yeast cells lacking Rub1, the yeast homolog of Nedd8 [8].

We then tested whether inhibition of polyubiquitin chain formation had an effect on the ability of Cul1 to bind the S5a subunit of the 19S proteasome *in vitro*. To do this, we included methylated ubiquitin, which is chemically modified to block all of its free amino groups and is therefore incapable of forming polyubiquitin chains, to the Cul1 *in vitro *translation reaction. We observed a change in the pattern of Cul1 bands that bind to GST-S5a in the presence of methylated ubiquitin (Figure 4C, compare lanes 2 and 4), with lower molecular weight forms of Cul1 binding GST-S5a in the presence of methylated ubiquitin. The fact that methylated ubiquitin changed both the Cul1 forms that bind to GST-S5a and the overall higher molecular weight Cul1 bands (Figure 4C, compare lanes 5 and 6) indicates that Cul1 is ubiquitylated *in vitro *and that ubiquitylated Cul1 associates with the S5a subunit of the 19S proteasome. It has been reported that isolated S5a has high affinity for ubiquitin while S5a complexed to the 19S proteasome does not [3]. However, our observation that modified forms of Cul1 co-purify with the 19S subcomplex (Figure 3) suggests that ubiquitylated Cul1 associates with the 19S proteasomes and not just free S5a.

### Inhibition of Cul1 polyubiquitylation *in vivo *alters the ability of Cul1 to bind S5a without affecting the stability of Cul1

**Figure 5:**
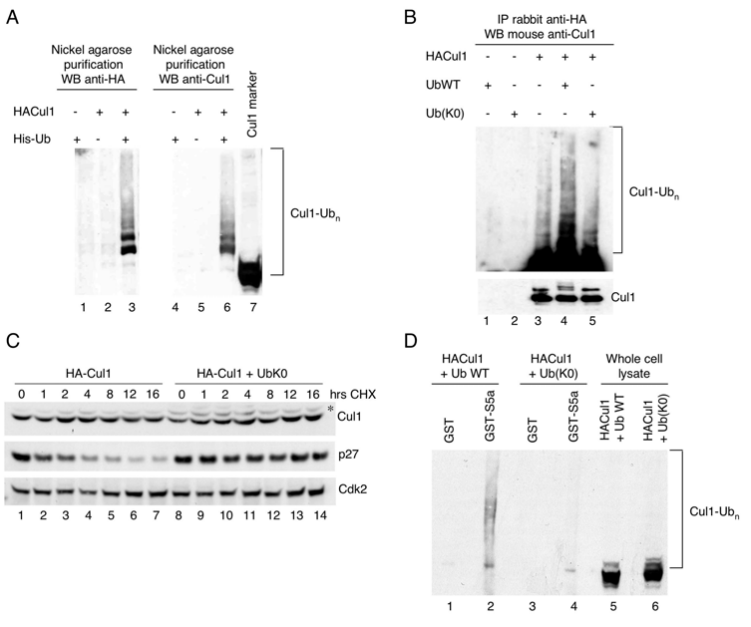
**Cul1 ubiquitylation promotes binding to the proteasome, but not degradation**. (A) 293T cells were transfected with His-tagged ubiquitin (lanes 1 and 4), HA-tagged Cul1 (lanes 2 and 5) or His-Ubiquitin and HA-Cul1 (lanes 3 and 6). Cell extracts were subjected to nickel agarose purification (lanes 1–6). Precipitates were analyzed by immunoblotting with an antibody against the HA tag (lanes 1–3) or an antibody to Cul1 (lanes 4–7). Lane 7 contains recombinant Cul1. The bracket on the left side of the images marks a ladder of bands >90,000 corresponding to ubiquitylated Cul1. (B) 293T cells were transfected with ubiquitin (UbWT) (lane 1), a Ub(K0) mutant (lane 2), HA-tagged Cul1 (lane 3), UbWT and HA-Cul1 (lane 4) or Ub(K0) and HA-Cul1 (lane 5). Cell extracts were immunoprecipitated with an anti-HA antibody. Precipitates were analyzed by immunoblotting with an antibody to Cul1 (lanes 4–7). The lower image represents a short exposure time and the upper image represents a long exposure time. The bracket on the left side of the images marks a ladder of bands >90,000 corresponding to ubiquitylated Cul1. (C) 293T cells were transfected with HA-Cul1 in combination with empty vector (lanes 1–7) or Ub(K0) mutant (lanes 8–14). Cells were incubated in the presence of cycloheximide (CHX) for the indicated times. Degradation of Cul1 was monitored by immunoblotting with an anti-HA antibody. The asterisk indicates the neddylated form of Cul1. (D) 293T cells were transfected with HA-tagged Cul1 plus wild-type ubiquitin (UbWT) (lanes 1–2 and 5) or Ub(K0) mutant (lanes 3–4 and 6). Lysates were subjected to a pulldown using GST alone (lanes 1 and 3) or GST-S5a (lanes 2 and 4). Lanes 5 and 6 contain whole cell lysate. Pulldowns and lysates were analyzed by immunoblotting with an antibody to Cul1. The bracket on the left side of the images marks a ladder of bands >90,000 corresponding to ubiquitylated Cul1.

We next confirmed that Cul1 is ubiquitylated *in vivo*. We cotransfected 293T cells with HA-tagged Cul1 and His-tagged ubiquitin, and lysates were subjected to a nickel agarose pulldown to purify ubiquitylated proteins through the histidine tag on ubiquitin. Immunoblotting of purified ubiquitylated proteins with an antibody to HA (Figure 5A, lanes 1–3) or to Cul1 (lanes 4–7) showed multiple higher molecular weight forms corresponding to ubiquitylated Cul1.

We then tested the effect of a mutant ubiquitin with all of its lysines mutated to arginines [Ub(K0)] on the levels and ubiquitylation status of Cul1. The Ub(K0) mutant, similarly to methylated ubiquitin used in the *in vitro *experiment, cannot form polyubiquitin chains. 293T cells were transfected with HA-tagged Cul1 in the presence of wild-type ubiquitin or Ub(K0) mutant, and extracts from transfected cells were immunoprecipated with an antibody to the HA tag on Cul1. Immunoprecipitates were analyzed by immunoblotting with an antibody to Cul1. High molecular weight species which we observed in the presence of wild-type ubiquitin disappeared when Ub(K0) mutant was cotransfected with Cul1 (Figure 5B, compare lanes 4 and 5), demonstrating that Ub(K0) mutant competes with endogenous ubiquitin to terminate ubiquitin chains on Cul1. Interestingly, Ub(K0) mutant did not alter the steady-state levels of Cul1 (Figure 5B, lower panel), while it has previously been shown to result in the accumulation of both p21 and cyclin E [18]. Additionally, no change in the steady-state levels of Cul1 was observed in the presence of Ub(K0) mutant by direct western blotting of these extracts with antibodies to Cul1 or the HA tag (data not shown). These results suggest that inhibition of polyubiquitylation of Cul1 does not affect the stability of Cul1.

To confirm this, we compared the half-life of Cul1 in the absence or presence of the Ub(K0) mutant. Cul1 did not exhibit a decrease in the rate of its degradation when polyubiquitin chain formation was blocked by overexpression of the Ub(K0) mutant (Figure 5C). In contrast, p27, an established substrate of the ubiquitin-proteasome pathway, was stabilized in the presence of the Ub(K0) mutant. It is known that K48-linked ubiquitin chains signal for proteasomal degradation, while other types of ubiquitin conjugates, including K63-linked chains, do not [5]. It is unclear at this whether Cul1 is polyubiquitylated via K48 or K63, and how, if it is polyubiquitylated via K48, it is recognized by proteasomes but not degraded. Prior studies have shown that the stability of Cul1 has been linked to its neddylation status in *Neuospora *[19] and *Drosophila *[20]. In these organisms, inactivation of the Cop9 signalosome (CSN), which deneddylates cullins, reduces the stability of Cul1. In contrast, in human cells, CSN-dependent deneddylation has no effect on Cul1 stability [21]. It is not yet clear why Cul1 is differentially regulated in these organisms.

Moreover, the mechanism for degradation of neddylated Cul1 has not yet been elucidated. We observed a minor increase in the levels of neddylated Cul1 in the presence of Ub(K0) but not a significant effect on Cul1 stability as was observed when CSN is inactivated in *Neuospora *[19] and *Drosophila *[20].

Next, we tested if inhibiting polyubiquitylation of Cul1 had an effect on the ability of Cul1 to associate with the S5a subunit of the 19S proteasome. Extracts from cells transfected with HA-tagged Cul1 in combination with wild-type ubiquitin or the Ub(K0) mutant were subjected to a pulldown with beads coated with GST alone or GST-S5a. Associated proteins were then analyzed for the presence of high molecular weight forms of Cul1 by immunoblotting with an antibody to Cul1. When polyubiquitylation was inhibited by expression of the Ub(K0) mutant, high molecular weight forms of Cul1 no longer bound to GST-S5a (Figure 5D, compare lanes 2 and 4). This result indicates that ubiquitylation of Cul1 *in vivo *is required for its interaction with the S5a proteasomal subunit. This may provide an additional mechanism for delivery of ubiquitylated substrates.

## Conclusion

We have shown that the association between SCF complexes and 26S proteasomes is conserved in higher eukaryotes. The Cul1 subunit, in particular, bound subunits of both the 19S and 20S subcomplexes. Interestingly, unmodified Cul1 was associated with 20S proteasomes, while high molecular weight forms of Cul1 associated with 19S proteasomes. We demonstrated that Cul1 is ubiquitylated in vivo, and that this modification affects the ability of Cul1 to bind to the S5a subunit of the 19S proteasome, without affecting the Cul1 levels. The association of Cul1 with the proteasome may help SCF complexes target ubiquitylated substrates for degradation.

## Methods

### Cell culture and transient transfections

293T and HeLa cells were maintained as previously described [22]. 293T cells were transfected by the calcium phosphate method.

### Biochemistry

Protein extraction, immunoprecipitations and immunoblots were performed as described [23]. Rabbit polyclonal antibody to Cul1, Cdk2 and p27 were previously described [24]. Monoclonal antibody to Skp1 was from BD Transduction Laboratories. Polyclonal antibodies to Roc1 and Skp2 were from Zymed Laboratories. Monoclonal antibodies to αlpha 2, αlpha 4 and αlpha subunits were from Affiniti Research. Monoclonal antibody to FLAG was from Sigma. Polyclonal antibody to HA was from Santa Cruz Biotechnology. Polyclonal antibody to 26S proteasomes was provided by Dr. George DeMartino. Polyclonal antibodies to S2 and S5a were provided by Dr. Martin Rechsteiner. Monoclonal antibody to αlpha6 was provided by Dr. Keiji Tanaka.

### GST pulldowns

GST, GST-S5a, GST-alpha 6 and GST-alpha 7 were purified using Glutathione Sepharose 4B (Amersham Biosciences) according to the manufacturer's instructions. Beads coated with GST alone or GST-tagged proteasomal subunits were incubated with *in vitro *translated Cul1 and binding buffer (0.05% Triton-X-100, 10% glycerol, 50 mM NaCl, 50 mM Na-HEPES pH 7.5) in a total volume of 0.5 ml. Beads were washed with binding buffer, and associated Cul1 was visualized by SDS-PAGE and autoradiography.

### *In vivo *ubiquitylation and degradation

For Cul1 ubiquitylation, 293T cells were cotransfected with HA-tagged Cul1 and His-myc-tagged ubiquitin. Extracts from transfected cells were prepared in lysis buffer including 100 μM NEM and 20 μM ubiquitin aldehyde. Lysates (0.5 ml volume) were denatured by boiling for 10 min in the presence of 1% SDS. Lysates were then incubated with 100 μl of 10% Triton X-100 and 400 μl lysis buffer on ice for 30 min prior to nickel agarose purification or immnoprecipitation. To measure protein half-lives, 293T cells cotransfected with HA-tagged Cul1 and wild-type ubiquitin or the Ub(K0) mutant were incubated in the presence of 100 μicrogram/ml cycloheximide (Sigma) diluted in 100% ethanol.

## Competing interests

M.P. is co-editor-in-chief of Cell Division but was not involved in the review process of this manuscript.

## Authors' contributions

JB performed the *in vivo *and *in vitro *immunoprecipitation and pull-down experiments and participated in the design of this study and drafting of the manuscript. AP carried out half-life experiments and participated in the design of this study. MP participated in the design of this study and drafting of the manuscript. GD purified proteasome particles. All authors read and approved the final manuscript.
